# Physiological accumulation of lipid droplets in the newborn liver during breastfeeding is driven by TLR4 ligands

**DOI:** 10.1016/j.jlr.2025.100744

**Published:** 2025-01-13

**Authors:** Wanderson Ferreira da Silva Júnior, Karen Marques de Oliveira Costa, Hortência Maciel Castro Oliveira, Maísa Mota Antunes, Kassiana Mafra, Brenda Naemi Nakagaki, Pedro Sérgio Corradi da Silva, Júlia Duarte Megale, Sarah Campos de Sales, Douglas Carvalho Caixeta, Mário Machado Martins, Robinson Sabino-Silva, Cristina Maria Pinto de Paula, Luiz Ricardo Goulart, Rafael Machado Rezende, Gustavo Batista Menezes

**Affiliations:** 1 Center for Gastrointestinal Biology, Departamento de Morfologia, Instituto de Ciências Biológicas, Universidade Federal de Minas Gerais, Belo Horizonte, Minas Gerais, Brazil; 2 Innovation Center in Salivary Diagnostics and Nanobiotechnology, Department of Physiology, Institute of Biomedical Sciences, Federal University of Uberlandia, Uberlandia, Minas Gerais, Brazil; 3 Laboratory of Nanobiotechnology, Institute of Biotechnology, Federal University of Uberlandia, Uberlandia, Brazil; 4 Laboratory of Nanobiotechnology, Institute of Genetics and Biochemistry, Federal University of Uberlandia, Uberlandia, Minas Gerais, Brazil; 5 Ann Romney Center for Neurologic Diseases, Brigham and Women’s Hospital, Harvard Medical School, Boston, MA, USA

**Keywords:** liver, newborn, lipid droplets, milk, TLR4, palmitic acid

## Abstract

The liver plays a central role in fat storage, but little is known about physiological fat accumulation during early development. Here we investigated a transient surge in hepatic lipid droplets observed in newborn mice immediately after birth. We developed a novel model to quantify liver fat content without tissue processing. Using high-resolution microscopy assessed the spatial distribution of lipid droplets within hepatocytes. Lugol's iodine staining determined the timing weaning period, and milk deprivation experiments investigated the relationship between milk intake and fat accumulation. Lipidomic analysis revealed changes in the metabolic profile of the developing liver. Finally, we investigated the role of Toll-like receptor 4 (TLR4) signaling in fat storage using knockout mice and cell-specific deletion strategies. Newborn mice displayed a dramatic accumulation of hepatic lipid droplets within the first 12 h after birth, persisting for the initial two weeks of life. This pattern coincided with exclusive milk feeding and completely abated by the third week, aligning with weaning. Importantly, the observed fat accumulation shared characteristics with established models of pathological steatosis, suggesting potential biological relevance. Lipid droplets were primarily localized within the cytoplasm of hepatocytes. Milk deprivation experiments demonstrated that milk intake is the primary driver of this transient fat accumulation. Lipidomic analysis revealed significant changes in the metabolic profile of newborn livers compared to adults. Interestingly, several highly abundant lipids in newborns were identified as putative ligands for TLR4. Subsequent studies using TLR4-deficient mice and cell-specific deletion revealed that TLR4 signaling, particularly within hepatocytes, plays a critical role in driving fat storage within the newborn liver. Additionally, a potential collaboration between metabolic and immune systems was suggested by the observed effects of myeloid cell-specific TLR4 ablation. This study demonstrates a unique phenomenon of transient hepatic fat accumulation in newborn mice driven by milk intake and potentially regulated by TLR4 signaling, particularly within hepatocytes.

Lipid droplets (LDs) have been extensively recognized as dynamic organelles with a multifaceted role in cellular physiology (1, 2). While their primary function remains energy storage through the sequestration of triglycerides, LDs contribute significantly to cellular homeostasis through multiple mechanisms (2, 3, 4). Notably, LDs may act as buffers against lipotoxic stress by sequestering excess fatty acids and interacting with mitochondria to facilitate fatty acid channeling for optimal β-oxidation and ATP generation (2, 5). Additionally, LDs are highly involved in cellular metabolism, serving as a source of fatty acids that drive catabolic mechanisms, including lipophagy and lipolysis (2, 3, 4). These multifaceted contributions underscore the vital role of LDs in maintaining cellular health and function (2, 3, 4).

Most cell types harbor a basal population of LDs for transient metabolic functions (2, 5); however, specific organs exhibit remarkable adaptations for extensive LD accumulation. Adipose tissue stands out as the preeminent fat storage depot, with mature adipocytes containing massive LDs that constitute the primary physiological energy reserve (6). Skeletal muscle, along with the heart, also harbors a significant population of LDs to fuel contractile activity (7, 8, 9). In the liver, however, the role of LDs is more complex since they can be also generated within hepatocytes via de novo lipogenesis using carbohydrates from the diet (10, 11). These newly synthesized fatty acids are subsequently esterified into triglycerides and deposited within LDs (12, 13). Furthermore, the liver also orchestrates the assembly of lipoproteins, intricate macromolecular complexes that serve as vehicles for transporting triglycerides and cholesterol throughout the body (14). This dual capacity for LD storage and generation elevates the liver to a central hub for whole-body lipid metabolism, making it critical for maintaining energy homeostasis (15). Disruptions in the finely tuned regulation of LD dynamics within the liver can have profound ramifications on energy balance, potentially leading to the development of metabolic disorders such as non-alcoholic fatty liver disease (NAFLD) (16, 17). Elucidating the intricate interplay between LDs and the liver metabolic machinery holds significant promise for the development of novel therapeutic strategies.

Interestingly, LDs are not only present in sessile conditions inside the cells; these organelles can also be secreted (18). For example, mammary gland epithelial cells produce and secrete large quantities of LDs in breast milk, providing a major source of energy for newborns (18). This process is tightly regulated by hormones and utilizes a unique apocrine mechanism (18). Within the rough endoplasmic reticulum, neutral lipids are synthesized and packaged into large LDs (5–15 μm) coated with a perilipin-2 (PLIN2) layer (18, 19). These LDs then undergo directed intracellular trafficking towards the apical plasma membrane for secretion (18, 20, 21, 22, 23, 24). Upon reaching their destination, a specific docking complex aids in their envelopment by the plasma membrane (18, 20, 21, 22, 23, 24). Finally, with the stimulation of oxytocin, surrounding myoepithelial cells contract, expelling the LDs into the milk lumen (18). These expelled LDs undergo a final transformation, acquiring a phospholipid monolayer and a coat of various proteins before being fully encased in a complete membrane bilayer (18). This final structure—the milk fat globule—is a signature component of mammalian milk (18, 23, 25).

Although the presence of LDs in breast milk is well-established (18, 19, 20, 21, 22, 23, 24, 25), a significant knowledge gap exists regarding the direct link between these milk LDs and fat storage within the developing livers of neonates. This intriguing question remains largely unexplored, particularly the potential mechanisms by which milk LDs might influence or interact with hepatic lipogenesis and lipolysis in the newborn liver, and also fuel immune cell development in the first moments of life outside the uterus (2). In fact, the signaling pathways that could trigger such effects on hepatic lipid metabolism are largely unknown. Unveiling these mechanisms could provide valuable insights into the early programming of lipid homeostasis in neonates and potentially inform future strategies for optimizing infant health. In this work, we describe hepatic lipid droplet accumulation during the postnatal period in mice, which even resembles the extent of tissue occupancy and individual lipid droplet sizes observed in NAFLD models. This conserved phenomenon is directly driven by milk intake and is fundamentally dependent on TLR4 downstream signaling in hepatocytes in collaboration with hepatic immune cells. To our knowledge, these data, characterized at this level for the first time, illuminate the complexity of an event inherent to liver and systemic development.

## Materials and methods

### Animals

This study utilized both male and female C57BL/6 wild-type adult mice sourced from the Centro de Bioterismo at the Universidade Federal de Minas Gerais. TLR4KO mice were obtained from The Jackson Laboratory. LysM-Cre-Tlr4-Flox and Alb-Cre-Tlr4-Flox mice were generated by crossing LysM-Cre and Alb-Cre mice with TLR4 f/f mice. All mice were genotyped and the efficacy of deletion guided by c-alb or lysm promoters was previously validated (26, 27, 28, 29). Animals of various ages (0 and 4 days; 1, 2, 3, and 4 weeks) representing the described genotypes were housed in a conventional specific pathogen-free facility at the Universidade Federal de Minas Gerais. Each acrylic cage (Alesco) housed five mice (two females per male per matrix) and featured a filtered air system. Mice received ad libitum access to Nuvilab autoclaved rodent chow and water, and a digitally controlled 12/12-h light/dark cycle was maintained. In pups-littermate separation experiments, newborns were immediately separated from moms after birth, and were maintained with a foster mom that was not able to breastfeed until the experiments (12 h maximum). Also, a separated set of pups were fed with skimmed milk (commercially available 0.1% of fat) or filtered water each 30 min for 12 h. All experimental procedures involving mice were conducted in accordance with international guidelines for animal care and received approval from the Animal Ethics Committee of the Universidade Federal de Minas Gerais (register number 172/2024).

### Confocal microscopy

This study utilized confocal microscopy imaging as previously described (30). Briefly, mice were first intravenously injected with 2 μg/Kg of anti-CD31 antibody conjugated to BD Horizon Brilliant™ Violet polymer (emission at 421 nm, BV421, clone 390, Becton Dickinson, BD) and, when necessary, anti-F4/80 Alexa Fluor 647 (2 μg/mouse, clone T45-2342, Becton Dickinson). After 20 min, mice were anesthetized with a Ketamine/Xylazine solution (60 mg/Kg and 15 mg/Kg, respectively; Syntec, São Paulo, Brazil). Following midline laparotomy, livers were excised, placed on a custom acrylic support optimized for confocal microscopy and administered 1 μg of Bodipy solution (Difluoro{2-[1-(3,5-dimethyl-2H-pyrrol-2-ylidene-N)ethyl]-3,5-dimethyl-1H-pyrrolato-N}boron, 1 mg/ml, Sigma-Aldrich, diluted in DMSO).

To spatially localize the boundaries of hepatocytes and accurately determine the location of lipid droplets (LDs), we developed a novel in vivo imaging strategy that involves labeling the cytoplasm of these cells with a fluorescent dye. Specifically, we utilized indocyanine green (ICG; i.v. 20 mg/kg or anti-ASGR1; BD Biosciences; 2 μg/mouse, clone 8D7). ICG is a tricarbocyanine dye that fluoresces upon excitation with near-infrared light at 806 nm. ICG is highly water-soluble and binds to β-lipoproteins, particularly albumin. This dye is widely used due to its selective uptake by the liver, where it is absorbed completely, metabolized, and excreted in bile in its unbound state. ASGR1 is a hepatocyte-selective marker (31). Confocal imaging was performed using an inverted Nikon Eclipse Ti microscope coupled with an A1R scanning head (Nikon). Hepatic fields and Bodipy-labeled lipid droplets were analyzed, and digital quantification was conducted using Volocity software (version 6.3; PerkinElmer) and NIS-Elements software (Nikon Instruments).

### Diet-induced liver steatosis

Mice were fed a control diet (10% calories from fat), and two different high-fat diets (40% calories from fat, Research Diets) for 16 weeks. HFDs had different common fat sources, including *trans*-fat (D0900301) and non-*trans*-fat palm oil (Primex-Z - D0900308). During the experimental protocols, mice were weighed weekly and received 100 g of chow twice a week. After 16 weeks of dietary challenge, mice were sacrificed by exsanguination after ketamine and xylazine anesthesia. Livers were prepared for confocal imaging as described previoulsy (30).

### Chow detection in gastric content

To assess chow intake in newborns and define the beginning of the weaning period, gastric content was obtained via midline laparotomy under anesthesia (day 4 onwards) and in adults. Approximately 100 μl of gastric content was collected and diluted 1:1 with Lugol's iodine solution for starch detection. Isolated milk samples served as negative controls (expected brown coloration), while chow itself diluted with Lugol's iodine served as a positive control (expected black solution). After a 5-min incubation at room temperature, samples were visually assessed for color change. Black coloration indicated a positive result for starch, potentially signifying chow intake, while samples retaining a brown color (similar to the milk control) were considered negative. To perform statistical analyses and ensure reproducibility, we utilized Chi-square test to evaluate the data. The results were categorized into 3 distinct groups to meet the non-zero value requirement of the Chi-square test: 1: Positive for chow; 0.5: Double positive (both milk and chow present); −1: Negative for chow. The analysis was conducted using an online Chi-square calculator (Social Science Statistics, socsistatistics.com). The test results indicated statistical significance, with *P* < 0.00001. Data were plotted in heatmap using Prism Graphpad 8.0.

### Lipid extraction and quantification

Lipids from tissue samples were extracted using a method adapted from previous works (32, 33), with some modifications. Liver tissues (n = 3/pool group) were weighed and homogenized with methylene chloride and methanol in a 0.05:1 (w/v) ratio. Deionized water was then added, and after vortexing, the samples were incubated for 30 min at room temperature. The homogenates were centrifuged at 3000 rpm for 20 min at 4°C. The organic phase was collected and dried in a Genevac™ miVac Centrifugal Concentrator. For analysis, the samples were resuspended in methylene chloride and methanol. Lipidomic analyses were performed using an Agilent Technologies 7890B gas chromatography-mass spectrometry (GC-MS) system, equipped with a PAL RSI 85 autosampler. A DB-5 column (30 m × 0.22 μm × 0.1 μm) with a flow rate of 1 ml/min was used in selected ion monitoring (SCAN) mode. The chromatographic parameters included a temperature ramp from 60°C to 250°C over 40 min. For this experiment, 10 μl of the samples were injected. MassHunter Qualitative v. 10.0 software was used to process the raw data. The 'molecular feature extraction (MFE)' tool was employed to extract the mass spectra and convert them to CEF format. The identification of lipid biomolecules was carried out using the NIST 2017 library.

### Lipidomic analysis

Agilent Mass Profiler Professional (MPP) v. B.13.1.1 software was utilized to filter and analyze the extracted molecular compounds. The filters applied included a minimum absolute abundance of 5,000 counts and all charges permitted. The analysis parameters were a retention time tolerance of 0.15 min and a mass window of 10 ppm. Only molecular compounds present in 100% of at least one group were considered for analysis. Statistical analyses of the lipidomic data were conducted using log2-transformed values. ANOVA, followed by Tukey's post-hoc test (*P* < 0.05), was used to identify which groups had altered lipid metabolites compared to the control group (adult developmental stage), with a fold change equal to or greater than 2.00.

### Statistical analyses

Statistical analyses and graph generation were performed using GraphPad Prism 6.0 software (GraphPad Software). All results are expressed as the mean ± standard error of the mean (SEM). Group comparisons were conducted using a one-way analysis of variance (ANOVA) followed by Dunnett's post-hoc test. Statistical significance was set at *P* < 0.05.

## Results

### Newborn livers exhibit a transient accumulation of lipid droplets, a phenomenon not observed in fetuses or healthy adults

**Fig. 1:**
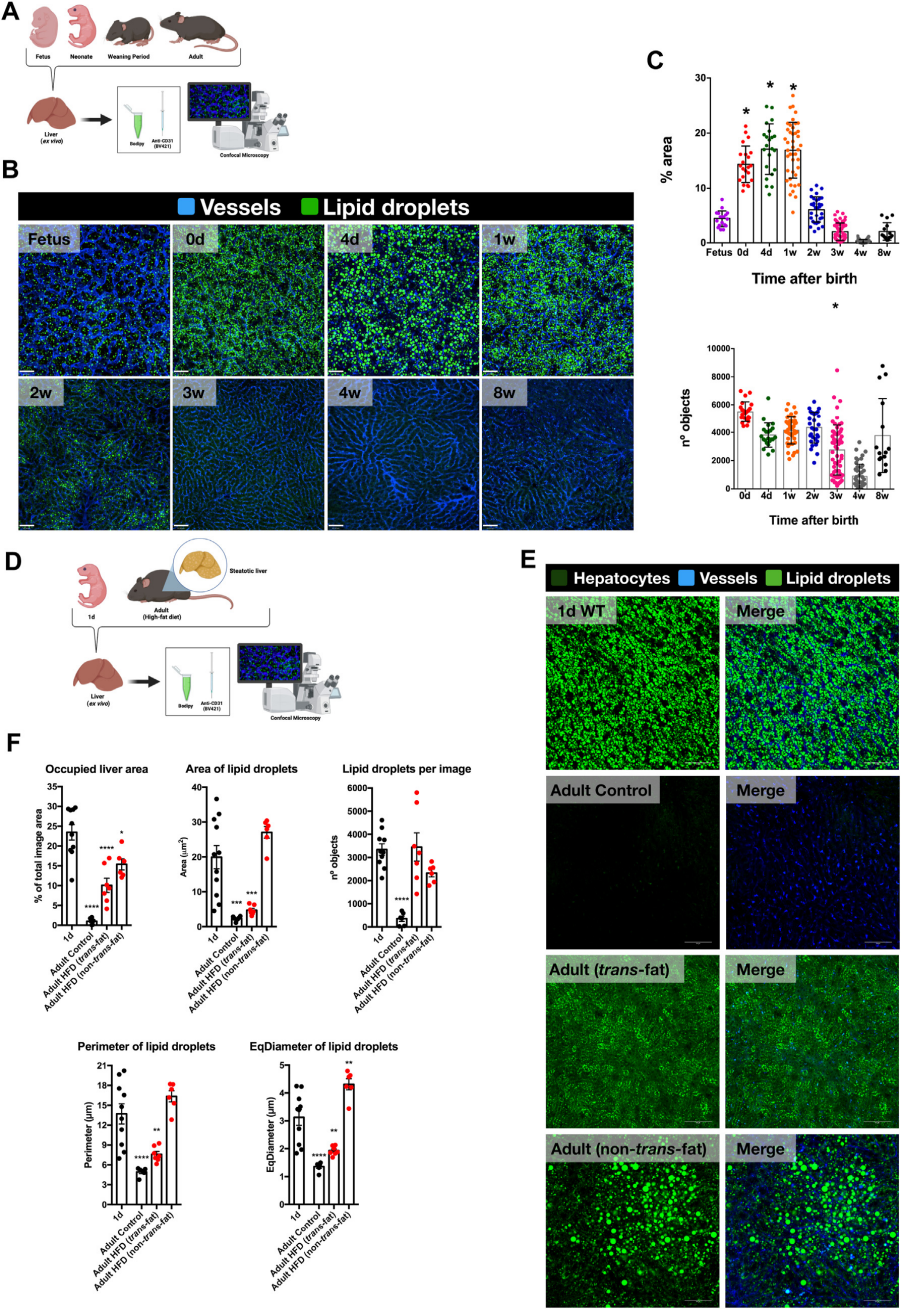
Newborn livers exhibit a transient accumulation of lipid droplets, a phenomenon not observed in fetuses or healthy adults. A: Livers from fetuses, neonates, weaning period mice, and adult mice were collected (ex vivo) after the intravenous administration of 2 μg/kg of anti-CD31 antibody conjugated to BD Horizon Brilliant™ Violet polymer (emission at 421 nm, BV421, clone 390, Becton Dickinson, BD) to visualize the hepatic sinusoids. On the Confocal Microscopy platform, Bodipy was applied to the liver surface to reveal the distribution of lipid droplets. B: Panel displaying the physiological accumulation of hepatic lipid droplets (stained in green with Bodipy) throughout the entire development of mice, from the fetal liver to adult mice: fetus, 0 days, 4 days, 1 week, 2 weeks, 3 weeks, 4 weeks, and 8 weeks of age. The hepatic sinusoids are shown in blue, stained by the anti-CD31 antibody. C: Quantification of the percentage of hepatic area occupied by lipid droplets throughout the entire kinetic, from fetuses to adult mice. The following graph displays the quantification of counted objects (lipid droplets) in fields of view throughout the same kinetic. D: The accumulation of hepatic lipid droplets in 1-day-old mice was compared to adult mice subjected to high-fat diets, *trans*-fat and non-*trans*-fat diets as described. The same method using anti-CD31 to mark the sinusoids and Bodipy for lipid droplets was applied. E: Panel displaying the distribution of lipid droplets within the liver of 1-day-old mice, adult control mice, adult mice subjected to a *trans*-fat diet, and adult mice subjected to a non-*trans*-fat diet. Lipid droplets are presented in green (stained by Bodipy), and the hepatic sinusoids are presented in blue (stained by the anti-CD31 antibody). F: Graphs presenting the quantitative analyses of the percentage of liver area occupied by lipid droplets, area of lipid droplets, number of lipid droplets per image, perimeter of lipid droplets, and diameter of lipid droplets between 1-day-old mice, adult control mice, adult mice subjected to a *trans*-fat diet and adult mice subjected to a non-*trans*-fat diet.

The liver plays a central role in fat storage, acting as a hub for not only dietary fat uptake but also its synthesis (15). Beyond simply storing excess fat from the diet, the liver can also generate fatty acids through a process called de novo lipogenesis (DNL) using carbohydrates or proteins as building blocks (10). This DNL activity can be significantly influenced by diet composition (11). There is a growing body of evidence that suggests that total calorie intake is a major factor in liver fat accumulation, with excessive calorie consumption regardless of macronutrient source promoting fat storage (11). However, our understanding of the potential for physiological liver fat accumulation during postnatal development remains incomplete, especially considering the dramatic shifts in nutrient sources that newborns experience (placental blood flow to breastmilk, then breastmilk to solid food) (34). To investigate this dynamic change in fat storage, we developed a new model for precise quantification of liver fat content without the need for chemical tissue processing (Fig. 1A). This model utilizes a fat-specific fluorescent dye (Bodipy) applied to the liver surface of mice previously injected with anti-CD31, an antibody that stains the liver's sinusoidal network. In fetal liver samples (third gestational week), we observed a scattered distribution of small lipid droplets primarily located outside the sinusoids (Fig. 1B). These droplets collectively occupied approximately 5% of the imaged area (Fig. 1C). However, a dramatic shift occurred just 12 h after birth. Day 0 newborn livers displayed a widespread and significant accumulation of lipid droplets across the field of view, occupying over 15% of the area (Fig. 1B, C). This substantial accumulation persisted on days 4 and 7 after birth, but began to decline by the second week (Fig. 1B, C). By the third week, coinciding with weaning period, lipid droplets were nearly undetectable (Fig. 1B). This pattern continued into adulthood. Interestingly, the total number of fluorescent objects in the field of view remained constant across all ages (Fig. 1C). This suggests that fat accumulation is quantitative rather than qualitative. In this sense, the number of “fat storage units” (likely lipid vesicles) appears to be consistent throughout life, but the amount of fat stored within each unit increases dramatically right after birth and then decreases after the third week (Fig. 1B, C). These findings demonstrate a surge in liver fat accumulation immediately following birth, that declines between the second and third week of life (Fig. 1B, C).

To assess the potential biological significance of this amount of neonatal hepatic fat accumulation, we compared it to established models of pathological fat storage, specifically non-alcoholic fatty liver disease (NAFLD) (35). To achieve this, we subjected mice to two different high-fat diets for a period of 16 weeks (Fig. 1D). A diet containing 40% *trans*-fat oil caused significant steatosis, characterized by widespread lipid accumulation within the liver (Fig. 1E). Interestingly, a diet rich in a non-*trans*-fat source also induced a severe form of steatosis, with over 15% of the liver area occupied by fat (Fig. 1E, F). Analysis of all parameters, including fat area and lipid droplet size and shape, revealed no significant difference between the fat accumulation observed in newborns and the high-fat diet-induced steatosis (Fig. 1F). This finding strongly suggests that the unique fat accumulation pattern in newborn livers has biological relevance and shares characteristics with established models of fatty liver disease.

### Lipid droplets in newborn livers are specifically stored within hepatocyte cytoplasm

**Fig. 2:**
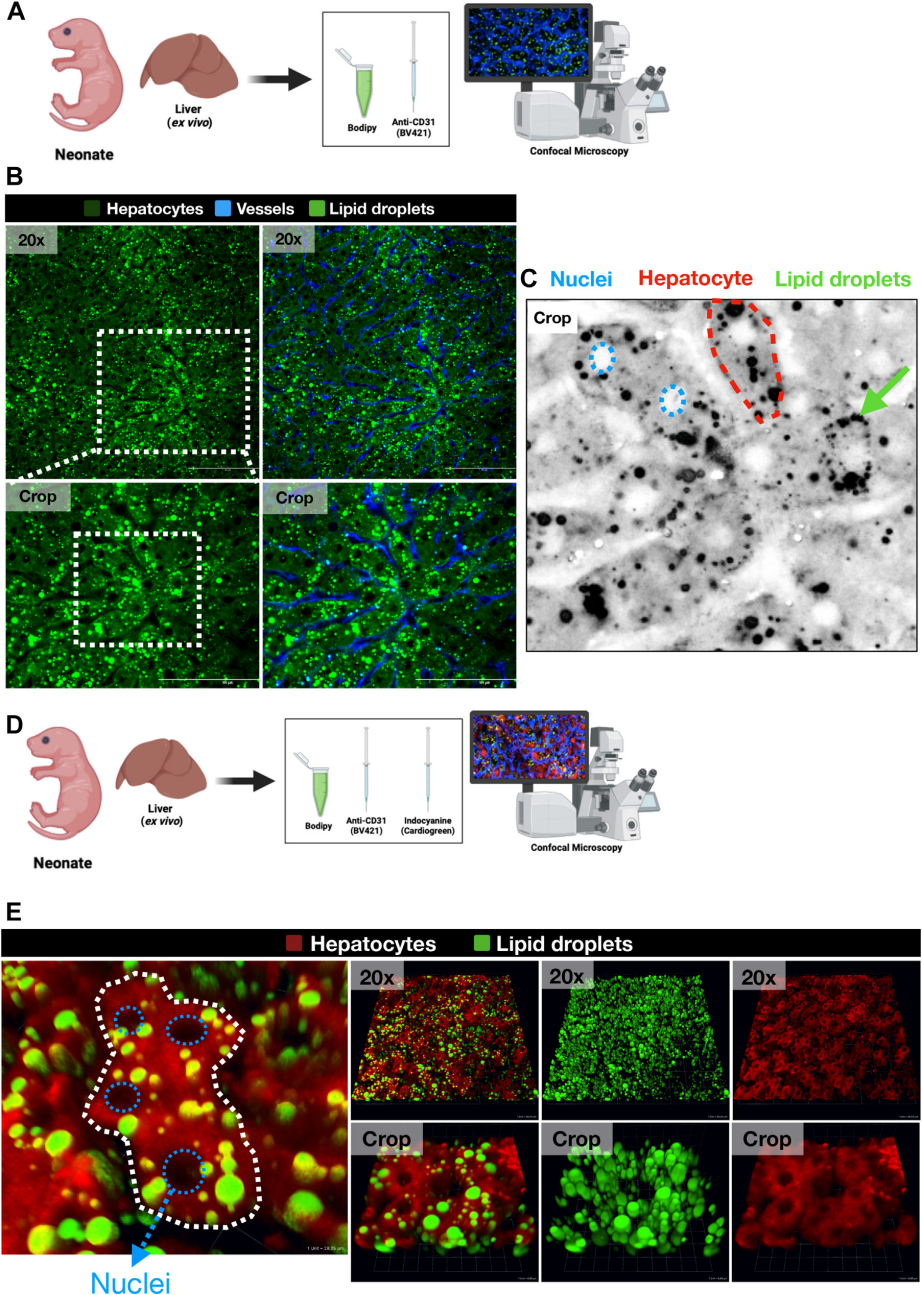
Lipid droplets in newborn livers are specifically stored within hepatocyte cytoplasm. A: Livers from neonate mice were collected (ex vivo) after intravenous administration of 2 μg/kg of anti-CD31 antibody conjugated to BD Horizon Brilliant™ Violet polymer (emission at 421 nm, BV421, clone 390, Becton Dickinson, BD) to visualize the hepatic sinusoids. Bodipy was applied to the hepatic surface to identify lipid droplets using Confocal Microscopy imaging. B: Panel of Confocal Microscopy images showing the apparent random distribution of lipid droplets in relation to hepatocytes. Lipid droplets are presented in vivid green (stained by Bodipy), hepatic sinusoids are presented in blue (stained by the anti-CD31 antibody) and hepatocytes are presented in darker green. C: A crop of confocal imaging evidencing the random disposition of lipid droplets in relation to hepatocytes in two dimensions. Lipid droplets appear to be disposed close to the hepatocyte membrane and the nuclear region, without a recognized pattern. D: For three-dimensional imaging reconstruction, livers from neonate mice were collected (ex vivo) after intravenous administration of Indocyanine (Cardiogreen, Sigma-Aldrich) to visualize the volume of hepatocytes. Bodipy was applied to the hepatic surface to identify lipid droplets. E: Panel of three-dimensional confocal imaging reconstruction of neonatal liver, evidencing the intracellular disposition of lipid droplets (stained in green by Bodipy) in relation to the volume of hepatocytes (shown in red by Indocyanine, Cardiogreen). Cytoplasmic lipid droplets are present throughout the entire hepatic field of view, close to the cell membrane or the nuclear region without a recognized pattern.

Lipid droplets are primarily cytoplasmic, scattered throughout the intracellular space in most cell types (2). However, in specialized cells like adipocytes, they can also accumulate near the nucleus (peri-nuclear) (6). This dual distribution reflects their potential roles in both general cellular metabolism and specialized functions (2). Having established hepatic lipid droplet accumulation in newborns, we next investigated their spatial distribution within hepatocytes and potential changes during development (Fig. 2A). To precisely define the boundaries of hepatocytes and identify the location of lipid droplets (LDs), we developed an innovative in vivo imaging approach that labels the cytoplasm of these cells with a fluorescent dye. We employed indocyanine green (ICG), a tricarbocyanine dye that emits fluorescence when excited by near-infrared light at 806 nm (36). ICG is highly soluble in water and binds to β-lipoproteins, particularly albumin. It is widely used due to its selective uptake by the liver, where it is fully absorbed, metabolized, and excreted in bile in its unbound form. Through in vivo three-dimensional reconstruction, we confirmed that more than 99% of lipid droplets in the liver co-localized with ICG fluorescence, verifying their strictly intracytoplasmic localization within hepatocytes (Fig. 2B, C). Some droplets were found near the plasma membrane, while others were observed circumscribing the nucleus (Fig. 2C). Three-dimensional rendering of liver samples (Fig. 2D) further revealed a subset of droplets in close proximity to the nuclear membrane, highlighting a widespread cytoplasmic localization of these fat stores (Fig. 2E). To determine whether lipid droplets (LDs) also accumulate in other hepatic cell types beyond hepatocytes, we also performed co-staining with Kupffer cell marker anti-F4/80. Three-dimensional reconstructions revealed that LDs were absent within Kupffer cells, indicating their predominant localization to hepatocytes (Supplemental Fig. 1 and Supplemental Movie 2). Collectively, our findings demonstrate a significant accumulation of hepatic lipid droplets within the first hours after birth, persisting for the initial two weeks of life.

### Analysis of gastric content indicates that C57BL/6 mice are fed exclusively on milk for the first 15 days after birth

**Fig. 3:**
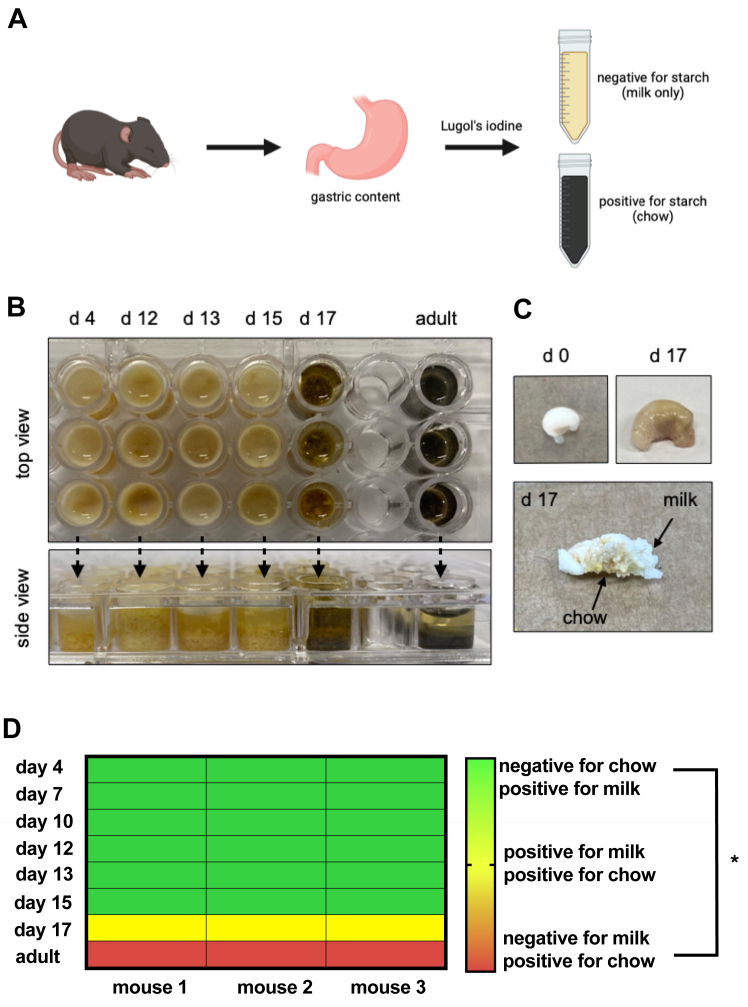
Analysis of gastric content indicates that C57BL/6 mice are fed exclusively on milk for the first 15 days after birth. A: The gastric content of neonate mice through adulthood was evaluated using Lugol’s Iodine stain to distinguish starch present in chow (absent in milk). Lugol’s Iodine (6% iodine in 4% potassium iodide) produces a yellowish-brown solution when mixed with milk only and a dark solution when mixed with chow diluted in water, indicating the presence of starch. B: Top and side views of pools containing solutions of gastric content from mice on day 4, day 12, day 13, day 15, day 17, and adults stained with Lugol’s Iodine. Darker solutions indicate the presence of starch from chow. C: Comparison of stomachs from mice on day 0 (milk spot) and day 17 (a more yellowish appearance). Upon dissecting the stomachs from day 17 mice, there is a presence of milk and chow content mixture. D: Heatmap displaying the results (negative or positive) of the starch reaction throughout the established time course: day 4, day 7, day 10, day 12, day 13, day 15, day 17, and adult. Only solutions from the gastric content of 17-day-old mice and adult mice reacted positively with Lugol’s Iodine, indicating starch from the chow. ∗ - *P* < 0.00001, chi-square test.

In the earliest days of life, newborn mice rely exclusively on their mother's milk for nourishment (37). However, pinpointing the exact start of weaning—the transition to solid food—proves to be a complex task (38, 39). Unlike humans with a well-defined weaning date, mice undergo a gradual shift from milk to a mixed solid and milk diet (38, 39, 40). This weaning period lacks a fixed timeframe and can vary between different mouse strains and even across experimental conditions (38, 39, 40, 41). To address this ambiguity and determine the precise window when solid food intake begins under our specific conditions, we developed a cost-effective model utilizing Lugol's iodine (LI) (Fig. 3A). This method leverages the unique property of LI (6% iodine in 4% potassium iodide) to stain starch, a component only present in solid food (chow) and absent in milk. We validated this approach by confirming that milk alone mixed with LI resulted in a yellowish-brown solution. As expected, LI produced a dark solution with a control mixture of chow diluted in water. Next, we repeated the protocol using gastric content collected from mice at different ages (Fig. 3B). Interestingly, Figure 3B shows that gastric content from days 4–15 tested negative for starch. However, gastric content from day 17 mice produced a clear brown solution, suggesting the initiation of solid food intake around this period (Fig. 3B). Direct observation of these stomachs confirmed the presence of chow mixed with milk, marking the beginning of weaning (Fig. 3C). Our findings indicate that, under these specific conditions, the weaning period commences around day 17 after birth (Fig. 3B–D). This timeframe coincides precisely with the observed reduction in hepatic lipid droplet accumulation (Fig. 1B, C).

### Milk intake immediately after birth induces acute lipid droplet accumulation within the newborn liver

**Fig. 4:**
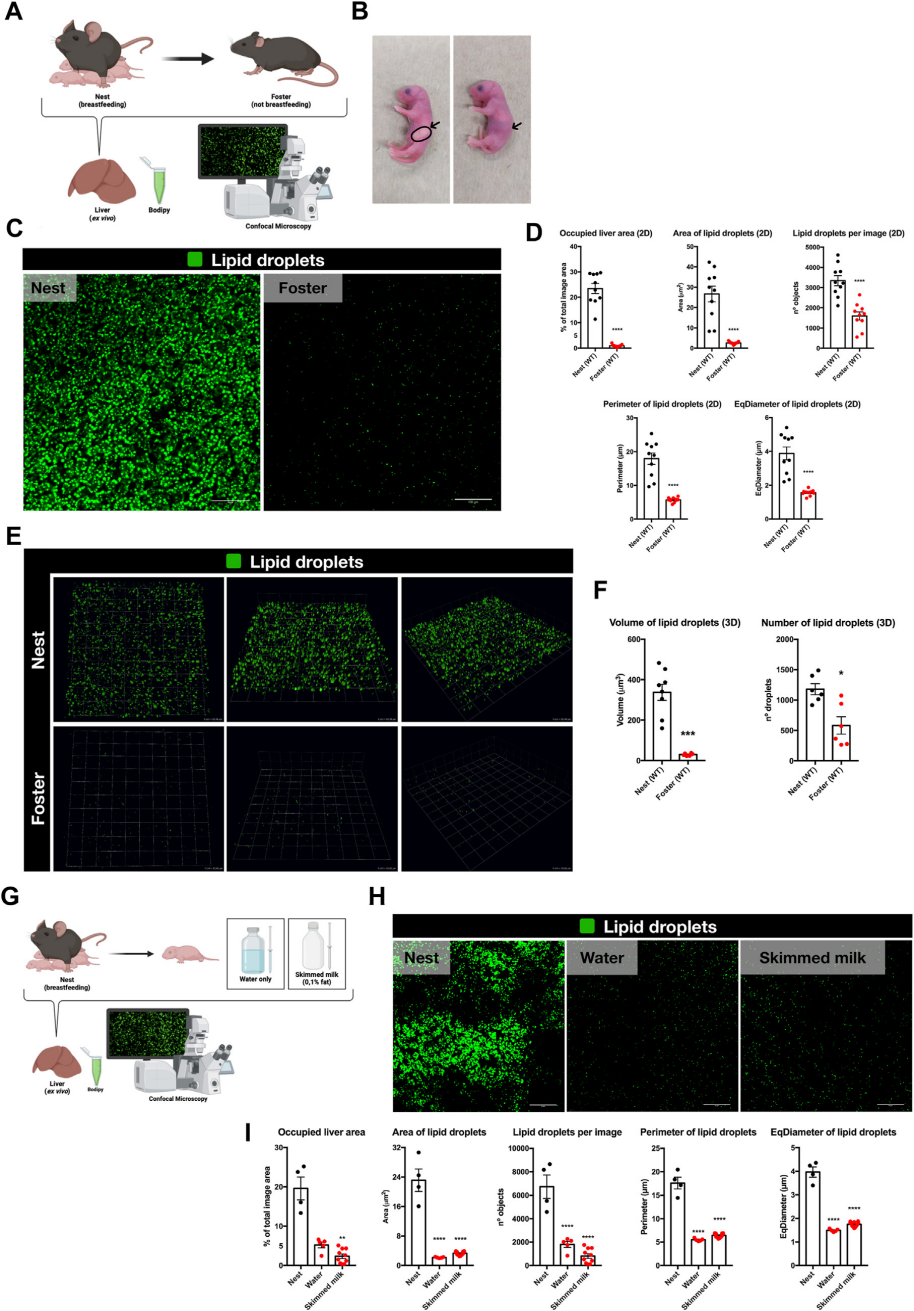
Milk intake immediately after birth induces acute lipid droplet accumulation within the newborn liver. A: a pregnant mouse was monitored throughout gestation until birth. The litter was then divided between the biological mother (Nest), capable of breastfeeding the pups, and a female mouse (Foster) that was not pregnant or breastfeeding but was kept in a nearby microisolator until receiving part of the litter. Livers from the neonates were collected 12 h after birth (ex vivo) and Bodipy was applied to the liver surface to identify the presence of lipid droplets during Confocal Microscopy imaging. B: Photos of newborn mice showing the milk spot within the stomach region of mice maintained with the biological mother (Nest) and the absence of the milk spot in mice maintained with the other female mouse (Foster). C: Panel of Confocal Microscopy images displaying the distribution of hepatic lipid droplets in newborn mice that were breastfed (Nest) and in mice that were not breastfed (Foster). Hepatic lipid droplets are shown in green (stained by Bodipy). D: Graphs showing the quantitative analyses of the percentage of hepatic area occupied by lipid droplets, area of lipid droplets, number of lipid droplets per image, the perimeter of lipid droplets, and eqdiameter of lipid droplets in newborn mice breastfed (Nest) and not breastfed (Foster). E: Three-dimensional reconstruction images displaying the distribution of lipid droplets within the liver of newborn mice breastfed (Nest) and not breastfed (Foster). Lipid droplets are shown in green (stained by Bodipy). F: Graphs presenting the quantitative analyses of the volume of lipid droplets and number of lipid droplets in three-dimensional reconstruction Confocal Microscopy images of livers from newborn mice breastfed (Nest) and not breastfed (Foster). G: a pregnant mouse was monitored throughout gestation until birth. Part of the litter was maintained with the biological mother (Nest), capable of breastfeeding the pups, while other newborn mice were subjected to water only or skimmed milk (0.1% fat). Livers from the neonates were collected (ex vivo) and Bodipy was applied to the hepatic surface to identify hepatic lipid droplets in confocal microscopy imaging. H: panel of Confocal Microscopy images showing the distribution of lipid droplets within the liver of newborn mice breastfed by the biological mother (Nest), newborn mice subjected to water only, and newborn mice subjected to skimmed milk (0.1% fat). Lipid droplets are presented in green (stained by Bodipy). I: Graphs presenting the quantitative analysis of the percentage of the occupied liver area by lipid droplets, area of lipid droplets, number of lipid droplets per image, the perimeter of lipid droplets, and the eqdiameter of lipid droplets in livers from newborn mice breastfed by the biological mother (Nest), newborn mice subjected to water only and newborn mice subjected to skimmed milk (0.1% fat).

The observed correlation between hepatic lipid droplet accumulation and the exclusive milk-based diet (Figs. 1B, C and 3) suggests that breastfeeding may be a major source of lipids driving fat storage in newborn livers. To investigate this hypothesis, we employed a separation method that minimized maternal deprivation stress, while avoiding pups to be fed with breast milk (Fig. 4A). In this pups-littermate separation experiment, newborns were immediately separated from moms after birth, and were maintained with a foster mom that was not able to breastfeed during the experimental period (12 h maximum). Newborn isolation effectively blocked milk intake, as evidenced by the absence of the "milk spot"—a whitish abdominal shadow caused by milk accumulation commonly seen in neonates (Fig. 4B). Livers from these unfed newborns displayed a dramatic reduction (>95%) in hepatic lipid droplet accumulation, appearing almost devoid of liver fat (Fig. 4C, D). This was confirmed by a significant decrease in fat area, number of lipid droplets per field, and all droplet dimensions (Fig. 4D). Three-dimensional analysis revealed a reduction not only in the number of lipid droplets but also in the overall volume of fat storage within the liver (Fig. 4E, F). To ensure the observed depletion wasn't due to dehydration, another group of mice received either water or skimmed milk (only 0.1% of fat) (Fig. 4G). Both treatments mirrored breast milk deprivation, leading to an 80%–90% reduction in hepatic lipid droplet accumulation (Fig. 4H, I). Additionally, all dimensional measurements of lipid droplets were significantly lower in the breast milk-deprived mice (Fig. 4I). Collectively, these data strongly suggest that during the pre-weaning period, the abundance of lipid droplets in newborn livers is almost entirely dependent on milk intake.

### Lipidomic analysis reveals major alterations of hepatic lipid constitution during postnatal development

**Fig. 5:**
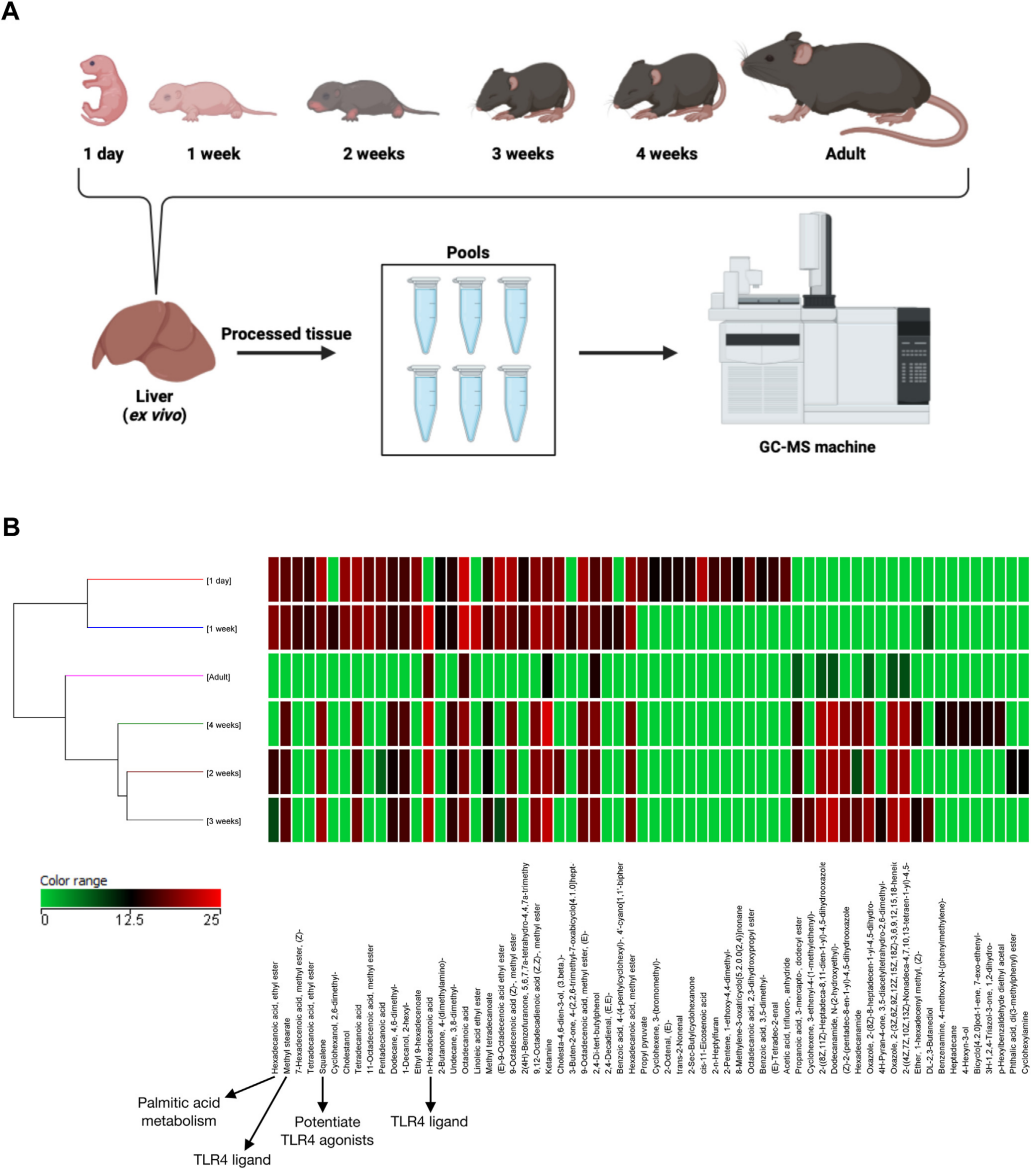
Lipidomic analysis reveals major alterations of hepatic lipid constitution during postnatal development. A: Livers from the entire kinetic series representing mouse development were collected: 1 day, 1 week, 2 weeks, 3 weeks, 4 weeks, and 8 weeks (adult). Total livers collected from mice were processed for evaluation by Gas Chromatography-Mass Spectrometry (GC-MS). B: Heatmap representing 64 compounds significantly differentiated between groups compared to adult mice (8 weeks). The green scale represents downregulation and the red scale represents upregulation in comparison. Some compounds are highlighted due to their putative relation with TLR4 downstream signaling and TLR4 agonists.

Following the characterization of lipid droplet dynamics and localization within the liver, we prepared samples for lipidomic analysis using gas chromatography-mass spectrometry (GC-MS) (Fig. 5A). Lipidomic analysis of liver tissue identified 787 metabolites following pre-processing, filtering, and alignment steps. Subsequent filtering based on a 100% presence criterion yielded a subset of 152 metabolites for further analysis. ANOVA test followed by Tukey's post hoc test was employed to identify significant changes in lipid metabolite abundance between different developmental stages (1 day, 1 week, 2 weeks, 3 weeks, and 4 weeks) compared to the adult control group (arrow in the heatmap) (Fig. 5B). This analysis revealed 64 metabolites with differential relative abundance (Fig. 5B). Notably, 7 compounds were found to be significantly decreased in the 1-day group compared to adults, while 39 metabolites showed a significant increase. Similar trends were observed in subsequent groups, with 6 and 32 metabolites exhibiting decreased and increased abundance, respectively, in the 1-week group compared to adults. The 2-weeks, 3-weeks, and 4-weeks groups displayed further increases in unique metabolites (28, 29, and 30, respectively) compared to the adult control. Data were clustered via their similarity, depicting the 64 most differentially expressed lipid metabolites identified through lipidomic (Fig. 5B). To facilitate data comparison obtained via CG-MS, metabolite abundance was normalized. The color scale represents relative metabolite abundance, with red indicating high abundance and green indicating low abundance. Each row in the heatmap corresponds to a specific metabolite identified in our analysis (Fig. 5B). Columns represent the relative abundance of metabolites within duplicate sample pools for each developmental stage (adult, 1 day, 1 week, 2 weeks, 3 weeks, and 4 weeks) (Fig. 5B). Taken together, these results confirm the significant difference in the metabolic profile of adults compared to the other developmental stages, especially in the pre-weaning period.

Following the identification of significant differences in lipid profiles between newborn and adult livers, we further explored the potential biological functions of these lipids. Public databases containing chemical structures (PubChem) were used to identify CAS registry numbers for the differentially abundant lipids. These numbers were then used to filter for known ligands of receptors relevant to liver biology. Interestingly, 4 of the highly abundant compounds in livers from 1-day-old to 2-week-old newborns were identified as putative ligands for Toll-like receptor 4 (TLR4) (Fig. 5B) (42, 43, 44, 45, 46, 47). As TLR4 expression on liver cells plays a multifaceted role, functioning as a sensor for microbial components and danger signals while also influencing liver metabolic function and potentially contributing to non-alcoholic fatty liver disease (NAFLD) (48, 49, 50, 51), this finding suggests these lipids may have significant biological effects within the liver.

### Collaborative TLR4 signaling in hepatocytes and myeloid cells drives lipid droplet accumulation within the newborn liver

**Fig. 6:**
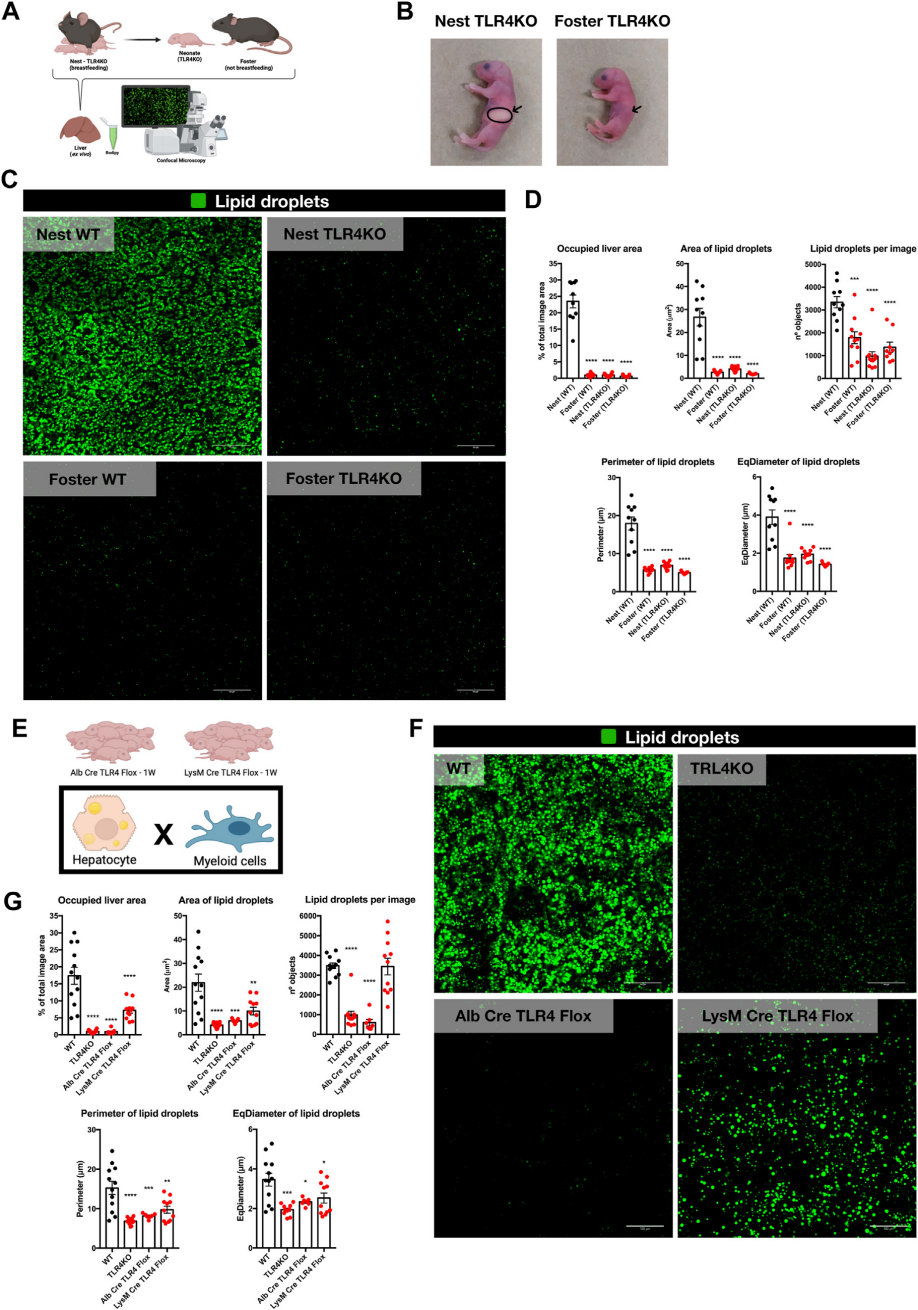
TLR4 signaling in hepatocytes, but not in myeloid cells, drives lipid droplet accumulation within the newborn liver. A: A pregnant TLR4KO mouse was monitored throughout gestation until birth. Part of the litter was maintained with the TLR4KO biological mother (Nest), capable of breastfeeding the pups, and part of the litter was maintained with a female mouse not able to breastfeed the pups (Foster). The livers from the newborn mice were collected (ex vivo) and Bodipy was applied to the liver surface to identify lipid droplets within the liver using Confocal Microscopy imaging. B: Photos of newborn TLR4KO mice showing the milk spot in the stomach region of TLR4KO mice that were breastfed (Nest) and the absence of a milk spot in TLR4KO mice that were not breastfed (Foster). C: Panel of Confocal Microscopy images displaying the distribution of hepatic lipid droplets within the livers of Wild Type mice that were breastfed (Nest WT), Wild Type mice that were not breastfed (Foster WT), TLR4KO mice that were breastfed (Nest TLR4KO) and TLR4KO mice that were not breastfed (Foster TLR4KO). Lipid droplets are shown in green (stained by Bodipy). D: Graphs presenting the quantitative analysis of the percentage of liver area occupied by lipid droplets, area of lipid droplets, number of lipid droplets per image, the perimeter of lipid droplets, and eqdiameter of lipid droplets in livers from newborn Wild Type mice that were breastfed (Nest WT), newborn Wild Type mice that were not breastfed (Foster WT), newborn TLR4KO mice that were breastfed (Nest TLR4KO) and newborn TLR4KO mice that were not breastfed (Foster TLR4KO). E: The same confocal microscopy imaging strategy was applied to evaluate the distribution of hepatic lipid droplets in the livers of newborn mice from Alb Cre TLR4 Flox mice, which have a conditional deletion of TLR4 in hepatocytes, and from LysM Cre TLR4 Flox mice, which have a conditional deletion of TLR4 in myeloid cells. F: Panel of Confocal Microscopy images displaying the distribution of hepatic lipid droplets within the livers of newborn Wild Type mice (WT), TLR4KO mice, Alb Cre TLR4 Flox mice, and LysM Cre TLR4 Flox mice. Lipid droplets are shown in green (stained by Bodipy). G: Graphs presenting the quantitative analysis of the percentage of liver area occupied by lipid droplets, area of lipid droplets, number of lipid droplets per image, the perimeter of lipid droplets, and eqdiameter of lipid droplets in livers from newborn Wild Type mice (WT), newborn TLR4KO mice, newborn Alb Cre TLR4 Flox mice, and newborn LysM Cre TLR4 Flox mice.

Given the abundance of putative TLR4 ligands identified in newborn liver lipid pools compared to adults (Fig. 5B), we investigated the role of TLR4 in hepatic lipid droplet formation (Fig. 6A). We compared lipid droplet accumulation in livers from wild-type and TLR4-deficient (TLR4^−/−^) newborn mice fed breast milk (Fig. 6C). Strikingly, TLR4^−/−^ day-old pups displayed a marked absence of lipid droplets compared to wild-type controls despite milk intake (Fig. 6C). These livers exhibited minimal fat accumulation and a significant reduction in all measured lipid droplet dimensions (Fig. 6C, D). These findings strongly suggest that TLR4 signaling, potentially triggered by liver-derived ligands (Fig. 5B), plays a critical role in driving fat storage within the neonatal liver. While hepatocytes are the primary functional units, the liver also houses a vast repertoire of myeloid immune cells, including Kupffer cells and dendritic cells (52, 53). These cells play vital roles in almost all biological responses (52). A full deletion of the TLR4 receptor would eliminate its function in both the hepatocytes and the immune cell population. This would create a complex scenario where both the metabolic and immune environments within the liver are altered. Such a broad deletion would make it difficult to pinpoint the exact site where TLR4 contributes to liver function. A more nuanced approach, such as cell-specific TLR4 knockout, appeared necessary to dissect its specific roles in hepatocytes and immune cells within the liver. To dissect the specific contribution of TLR4 in different liver cell types, we employed a Cre-lox deletion system to generate two novel mouse strains (Fig. 6E). The first strain combined mice harboring floxed TLR4 alleles (TLR4 f/f) with mice expressing Cre recombinase under the control of the albumin promoter (Alb-Cre) (Fig. 6E) (54, 55). This promoter drives Cre expression specifically in hepatocytes, leading to targeted deletion of TLR4 in these cells. A similar strategy was used to generate a second strain with selective TLR4 ablation in myeloid cells. Here, TLR4 f/f mice were crossed with LysM-Cre mice, where Cre expression is driven by the lysozyme M promoter, which is highly active in myeloid cells (56). Further analysis of lipid droplet formation in day-old pups revealed distinct effects of TLR4 deletion in different cell types (Fig. 6F). While myeloid cell-specific TLR4 ablation (LysM-Cre TLR4 f/f) led to a significant reduction in fat accumulation (∼50% decrease in the lipid-occupied area) compared to wild-type controls, the number of lipid droplets remained unchanged (Fig. 6F, G). In contrast, hepatocyte-specific TLR4 deletion (Alb-Cre TLR4 f/f) resulted in a much more profound effect (Fig. 6F, G). These pups exhibited an almost complete absence of hepatic fat content following breast milk intake (Fig. 6F). This was confirmed by a massive reduction in both the area occupied by lipid droplets and their absolute number within the liver, closely mirroring the effects observed in complete TLR4 knockout mice (TLR4−/−) (Fig. 6F, G). These findings suggest that during the initial milk intake period, a collaborative network between metabolic and immune systems might be involved in fat accumulation. However, TLR4 signaling specifically in hepatocytes appears to play a more critical role in driving fat storage within the newborn liver.

## Discussion

We have previously shown that the newborn liver exhibits significant differences compared to its adult counterpart, reflecting both the distinct nutritional demands of the early postnatal period and the immature immune system (34, 57). Unlike adults who can access a wider range of dietary sources, newborns rely solely on breast milk, which is rich in fats and carbohydrates (37, 58, 59). This necessitates adaptations within the liver to efficiently store and utilize these incoming nutrients. One such adaptation was revealed in this work since we showed a transient accumulation of lipid droplets during the first two weeks of life. Additionally, the developing immune system in newborns has not yet been fully exposed to the vast array of environmental antigens. This limited exposure could influence the role of the liver in immune regulation (34, 57). Notably, our findings suggest a potential role for TLR4 signaling, a key player in the innate immune response, in regulating fat storage within the newborn liver (Fig. 6). This study sheds light on a unique phenomenon—the transient accumulation of hepatic lipid droplets observed in newborn mice immediately after birth.

Our data reveal a surge in fat storage within the liver during the first two weeks of life, coinciding precisely with the period of exclusive milk feeding (Figs. 1B, C and 3). Microbiota colonization in mice becomes stable during the weaning period, typically around 12–14 days post-birth (60). However, it is clear that microbiota begins to colonize as soon as the mice leave the uterus. In our experiments, we observed lipid accumulation in the liver within an hour of the first milk intake, which makes it highly unlikely that the incipient microbiota exposure at this stage would be responsible for inducing such lipid accumulation through microbiome-related stimuli. While an experiment involving germ-free mice could provide insight, it is well-established that their immune response differs significantly from that of conventional mice, which would limit the interpretation of the results. An important observation is that the extent of fat accumulation in the adult liver, where microbiota colonization is stable, mirrors that seen in the fetal liver, which lacks microbiota. This comparison strongly suggests that the microbiota does not significantly contribute to lipid accumulation in the liver. Furthermore, our previous findings indicate that liver metabolic pathways mature around the time of weaning (34), which likely explains the dynamics of lipid droplet appearance and disappearance in the liver. Therefore, we propose that any potential influence of the microbiota would be minimal, with the maturation of metabolic processes being the more likely driver of this phenomenon. This lipid accumulation diminishes by the third week, coinciding with the onset of weaning (Figs. 1B, C and 3). This timing suggests a possible connection between the dietary transition from placental blood supply to breast milk and the observed changes in hepatic fat storage.

Interestingly, the observed fat accumulation in newborn livers shares characteristics with established models of pathological steatosis, such as non-alcoholic fatty liver disease (NAFLD) (Fig. 1E, F). Analysis revealed comparable total fat content and droplet size in newborns compared to mice fed high-fat diets (Fig. 1E, F). However, unlike NAFLD, which is considered a detrimental health condition, this process in newborns appears to be a physiological adaptation crucial for the early postnatal period (Fig. 1B). This distinction highlights the importance of understanding the context in which fat accumulation occurs within the liver. Further investigation revealed that the lipid droplets were primarily localized within the cytoplasm of hepatocytes (Fig. 2), suggesting their potential role in cellular metabolism. The specific functions of these cytoplasmic lipid droplets remain to be elucidated. However, their intracellular location suggests they may serve as a readily available energy source or play a role in cellular signaling pathways critical for early development (2, 61, 62). Importantly, milk intake seems to be the primary driver of this fat accumulation. Newborns deprived of breast milk exhibited a dramatic reduction in hepatic lipid droplets, highlighting the critical role of milk as a source of fat for the developing liver (Fig. 4). This finding underscores the importance of maternal milk in providing the necessary building blocks for proper liver function and development during this critical window.

Further lipidomic analysis revealed significant changes in the metabolic profile of newborn livers compared to adults, particularly during the pre-weaning period (Fig. 5). This suggests a dynamic metabolic landscape during this critical developmental window, potentially reflecting the rapid growth and organ maturation processes occurring at this time. Notably, we identified several highly abundant lipid compounds in newborn livers that potentially act as ligands for Toll-like receptor 4 (TLR4) (Fig. 5B). TLR4 is a pattern recognition receptor known to play a role in the innate immune response (63, 64). The identification of these potential TLR4 ligands (Fig. 5) (42, 43, 44, 45, 46, 47) suggests a possible novel link between immune and metabolic systems in the regulation of fat storage within the newborn liver. The co-occurrence of fat storage and hematopoiesis within certain tissues presents an intriguing biological phenomenon (65). Organs like the yolk sac in developing embryos and bone marrow in adults serve as primary sites for hematopoiesis (65, 66). Interestingly, these same tissues also harbor significant fat reserves (65, 67). This spatial overlap suggests a potential link or even a coordinated regulation between these seemingly disparate processes. In fact, we have shown that neonatal livers may serve as a key site for extra-uterine maturation of immune cells, since a large population of immune cell precursors and immature cells are present within the liver up to the first week after birth (34, 57). In this sense, fat stores could provide a readily available energy source for the high metabolic demands of hematopoietic cells, a premise that still deserves further investigation.

The observation of lipid attenuation in the liver at two weeks, despite continued breastfeeding, is an intriguing phenomenon that aligns with the dynamic metabolic adaptations during early postnatal development. Recent data have demonstrated that the neonatal liver undergoes a metabolic switch from glycogenolysis to fatty acid oxidation and gluconeogenesis, ensuring glucose homeostasis in neonates (68). This metabolic flexibility may provide a framework for understanding the observed reduction in hepatic lipid stores. One plausible explanation is that lipids are utilized to meet the increasing energy demands of rapid neonatal growth, as glycogen reserves become less dominant in energy supply. Additionally, the temporal patterns observed in our lipidomics suggest that the attenuation of metabolite levels corresponds to maturation of metabolic pathways and shifts in energy substrate utilization. The progressive reduction in metabolites at 1 and two weeks could reflect a transition in systemic energy regulation, possibly influenced by changes in milk composition and the neonate’s developing metabolic autonomy. While specific TLR4 ligands persist beyond 3 to 4 weeks, as shown in our data, their continued presence appears to have a limited role in sustaining lipid accumulation in the liver. This dissociation may indicate that the neonatal liver gradually reduces its reliance on TLR4-mediated signaling as other regulatory mechanisms, such as hormonal control and enzymatic shifts, take precedence. These findings are consistent with the adaptive metabolic remodeling reported recently (68), where the liver functions as a critical hub for converting lipid-derived metabolites into glucose to support homeostasis. Further studies are needed to elucidate the interplay between immune signaling, hormonal regulation, and enzymatic activity in driving these developmental changes. Nonetheless, our observations highlight the central role of the liver in managing neonatal energy balance through dynamic metabolic transitions.

Milk contains a diverse array of molecules, identified through various methods, that can influence biological activity by modulating immune-related pathways (59, 69). These compounds may influence infants' immune signaling, promoting immune protection and maturation (59, 69). Milk is becoming increasingly recognized as a critical factor in early innate immune system development (69, 70, 71) since several TLR-related pathways could be found within its components. In fact, soluble TLR signaling pathway inhibitors with anti-inflammatory effects via TLR2 and TLR4 have been identified (72, 73, 74), along with glycoproteins with anti-inflammatory effects via TLR4 (75, 76) and peptides/oligosaccharides with anti-inflammatory effect via TLR3, TLR4, and TLR7 (70, 77, 78). In our liver tissue lipidomics analysis, several identified compounds overlap with those described in studies of human milk composition. For instance, n-hexadecanoic acid (C16H32O2, CAS ID 57-10-3), also known as palmitic acid, was found to be upregulated during the early weeks of development (Fig. 5B, Supplemental Table 1). This compound, a major and stable fatty acid in human milk, constitutes approximately 25% of its total fatty acid content, as reported in multiple studies (79, 80, 81). The significance of palmitic acid in human milk is further highlighted by research on infant formulas, which examines how its presence and structural positioning influence optimal fat absorption in infants (82). However, the notion that palmitic acid functions as a ligand for TLR4 is met with both supporting and conflicting evidence in the literature (43, 44, 83). Beyond palmitic acid, there are also concrete findings of some of our lipidomic compounds in the milk literature, such as squalene (84), stearic acid (85) (octadecanoic acid, CAS ID = 57-11-4), and pentadecanoic acid (86), reinforcing that several of the lipids we identified in liver tissue have already been found in milk in previous studies.

To investigate the potential role of TLR4 signaling, we utilized TLR4-deficient mice (Fig. 6). Strikingly, these mice displayed a near-complete absence of hepatic fat accumulation compared to wild-type controls despite milk intake (Fig. 6C, D). This finding suggests a critical role for TLR4 signaling in driving fat storage within the newborn liver. It is possible that TLR4 signaling is activated by specific components within breast milk, leading to downstream pathways that promote fat storage in hepatocytes. Further research is needed to elucidate the specific mechanisms by which TLR4 signaling regulates this process. Interestingly, further data using cell-specific TLR4 ablation revealed a potential cooperative network between metabolic and immune systems (Fig. 6E–G). While myeloid cell-specific TLR4 ablation also reduced fat accumulation, the effect was less pronounced compared to hepatocyte-specific deletion (Fig. 6F, G). This suggests that TLR4 signaling within hepatocytes plays a more central role in regulating hepatic fat storage during the early postnatal period (Fig. 6F, G). The observation that myeloid cell-specific ablation still has some effect suggests a potential collaboration between immune and metabolic systems (Fig. 6F, G). However, the more pronounced effect of hepatocyte-specific deletion highlights the primary role of TLR4 signaling within hepatocytes in driving this process (Fig. 6F, G).

In conclusion, this study unveils a unique physiological adaptation in the developing liver. We observed a transient surge in fat storage driven by milk intake and potentially regulated by TLR4 signaling, particularly within hepatocytes. This finding highlights the importance of understanding the context-dependent nature of fat accumulation within the liver. Future investigations should focus on elucidating the specific mechanisms by which TLR4 signaling in hepatocytes regulates fat storage and the potential downstream targets involved. Additionally, exploring the long-term consequences of this transient steatosis on later health outcomes, such as susceptibility to metabolic disorders, could consist in a valuable avenue for future research.

## Data availability

All data related to lipidomic are provided in Supplemental Table 1.

## Supplemental data

This article contains supplemental data.

## Conflict of interest

The authors declare that they have no conflicts of interest with the contents of this article.

## Supplementary data

Supplemental Movie 2

Analysis Lipidomics - Anova
