# Supplementary material for: Physiological accumulation of lipid droplets in the newborn liver during breastfeeding is driven by TLR4 ligands
Source: J Lipid Res. 2025 Jan 13;66(2):100744. doi: 10.1016/j.jlr.2025.100744 (PMC11849619; doi:10.1016/j.jlr.2025.100744)
Supplement: Supplemental Figure S1 [file mmc1.pdf]

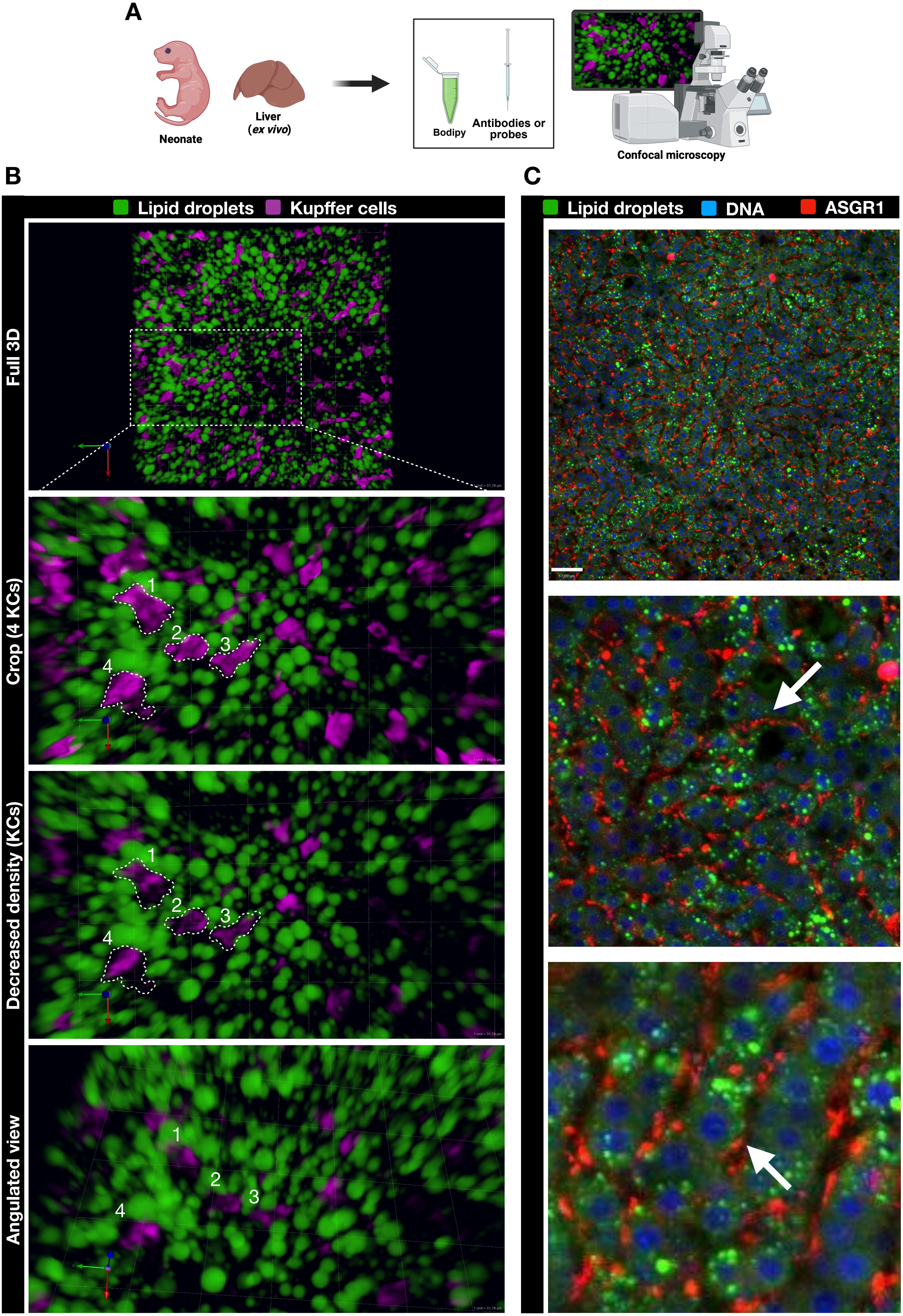
A
Neonate
Liver
(ex vivo)
Bodipy
Antibodies or probes
Confocal microscopy
B
Lipid droplets
Kupffer cells
Full 3D
Crop (4 KCs)
Decreased density (KCs)
Angulated view
1
2
3
4
C
Lipid droplets
DNA
ASGR1
