# Supplementary material for: Physiological accumulation of lipid droplets in the newborn liver during breastfeeding is driven by TLR4 ligands
Source: J Lipid Res. 2025 Jan 13;66(2):100744. doi: 10.1016/j.jlr.2025.100744 (PMC11849619; doi:10.1016/j.jlr.2025.100744)
Supplement: Supplemental Figure S2 [file mmc2.pdf]

A

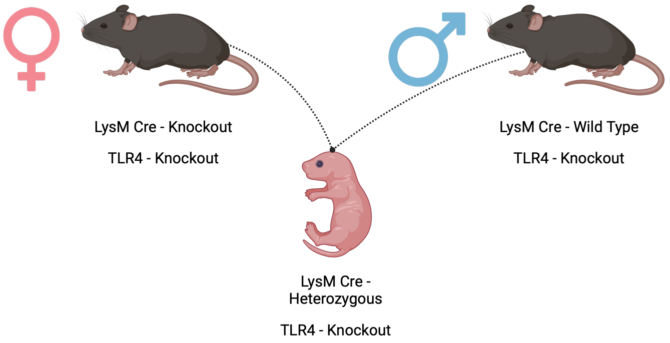


B

**LysM Cre TLR4 Flox**

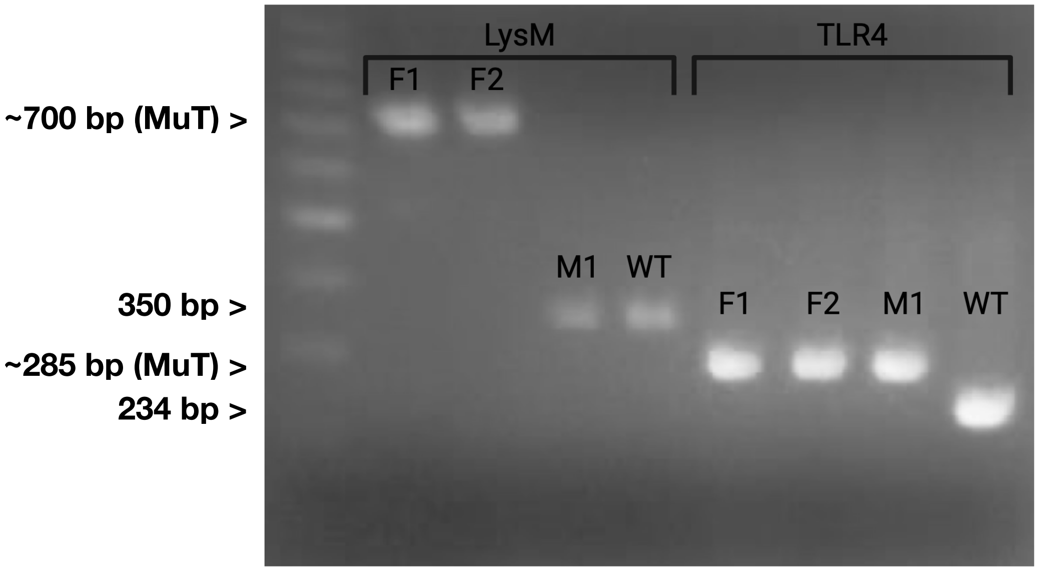


F1: female 1; F2: female 2; M1: male 1; WT: wild type; MuT: mutant.

C

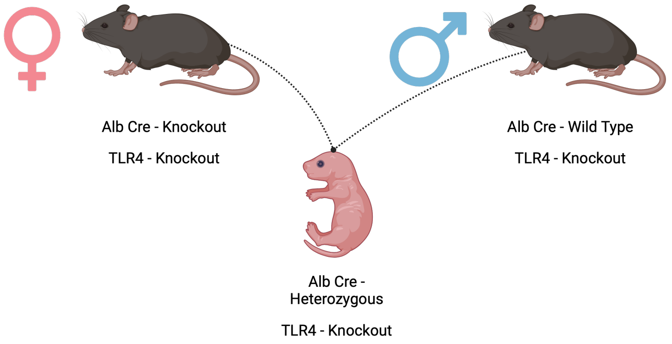


D

**Alb Cre TLR4 Flox**

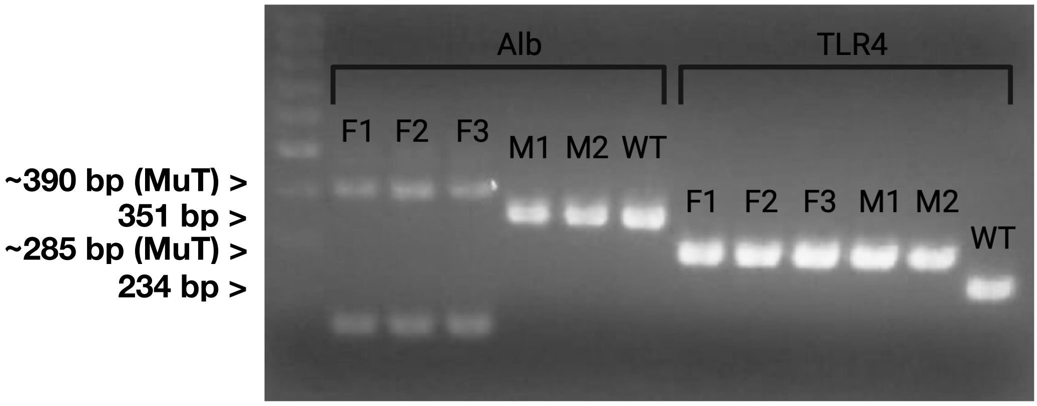


F1: female 1; F2: female 2; F3: female 3; M1: male 1; M2: male 2; WT: wild type; MuT: mutant.
